# Embedding Engineers in Clinical Pulmonology: Toward Earlier Clinical–Technological Integration

**DOI:** 10.3390/bioengineering13080873

**Published:** 2026-07-28

**Authors:** Manuel Casal-Guisande, María Torres-Durán, Alberto Fernández-Villar

**Affiliations:** 1 Department of Design in Engineering, University of Vigo, 36208 Vigo, Spain; 2 NeumoVigo I+i Research Group, Galicia Sur Health Research Institute (IIS Galicia Sur), SERGAS-UVIGO, 36312 Vigo, Spain; jose.alberto.fernandez.villar@sergas.es; 3 Centro de Investigación Biomédica en Red, CIBERES ISCIII, 28029 Madrid, Spain; 4 Pulmonary Department, Hospital Álvaro Cunqueiro, 36312 Vigo, Spain; 5 Department of Functional Biology and Health Sciences, University of Vigo, 36310 Vigo, Spain

Biomedical innovation often emerges from the interaction between clinical needs and technological expertise. However, this interaction does not always lead to solutions that can be readily adopted in real clinical settings. In hospitals, clinical engineering plays an essential role in ensuring that medical technologies are safe, reliable, and properly managed [1,2]. In this context, closer integration of engineering expertise within selected clinical environments may help bridge the gap between clinical needs, technological development, and practical implementation.

Pulmonology is a useful field in which to consider this form of integration. Respiratory medicine is increasingly shaped by the interaction between clinical assessment, longitudinal monitoring, digital health, and AI-based tools [3,4]. In this setting, the value of technology depends not only on how well it performs, but also on how naturally it fits into clinical workflows and decision-making. Many opportunities for innovation therefore do not begin as explicit technical requests. They often arise from everyday clinical challenges, such as diagnostic delays, inefficient pathways, heterogeneous patient profiles, underused routine data, or uncertainty in patient prioritization.

This is where embedded engineering may be useful. In this Editorial, embedded engineering refers to the stable presence of engineers within clinical departments, not as substitutes for clinicians or clinical engineers, but as part of a shared clinical–technological environment. This model should not be understood as a universal requirement for biomedical innovation. Many valuable AI and bioengineering studies are successfully developed through external or multidisciplinary collaborations. The argument is more specific: in departments with high technological complexity, large volumes of clinical data, and active translational research, proximity to clinical routines may help engineers identify relevant needs, understand practical constraints, and anticipate implementation barriers at an earlier stage.

Recent literature in respiratory medicine reinforces this point. AI and data-driven methods are increasingly being explored not only to improve diagnostic performance, but also to support patient stratification, prioritization, quality assessment, and clinical decision-making. In sleep-related disorders, recent reviews have described the growing use of AI models for diagnostic automation and pre-test detection, while also emphasizing the need for rigorous validation before clinical integration, particularly when multimodal clinical data are involved [5,6]. This message is especially relevant to obstructive sleep apnea, where high demand for sleep studies, limited diagnostic capacity, and patient heterogeneity have encouraged the development of tools for screening, prioritization, and decision support before sleep testing [7,8,9].

Similar challenges arise in other areas of respiratory medicine. In spirometry, AI-based approaches have been reviewed as potential tools for quality evaluation, a task closely linked to data reliability and real-world clinical performance [10]. In chronic obstructive pulmonary disease, routinely available clinical and social information has been explored to support patient characterization, prognostic assessment, and decision-support strategies [11,12]. In rare respiratory diseases such as alpha-1 antitrypsin deficiency, unsupervised learning has also been used to describe clinical heterogeneity and identify patient profiles [13]. These examples should not be interpreted as evidence that embedded engineering, by itself, improves clinical outcomes. Rather, they illustrate a more modest but relevant point: close interaction between clinicians and engineers can help transform routine clinical problems into structured, methodologically sound, and translationally oriented research questions.

The contribution of embedded engineers may therefore extend well beyond algorithm development. Their role can include problem formulation, methodological design, data governance, implementation planning, and bidirectional education. Working close to clinical practice, engineers may be better placed to judge whether available data are clinically meaningful, whether a model addresses a real need, and whether a technically promising solution can be incorporated into existing workflows. At the same time, clinicians who engage with engineering reasoning may gain a clearer understanding of what AI, modeling, and digital health can—and cannot—offer.

However, this model also raises an important professional issue. Engineers embedded in clinical departments should not be viewed only as technical assistants, data analysts, or project-based collaborators. If this role is to become sustainable, it requires clear responsibilities, appropriate supervision, authorship recognition, access to funding, training pathways, ethical and data-governance frameworks, and institutional support. This is not yet an established career model, but it is an organizational question that hospitals pursuing sustained clinical–technological innovation may need to address.

Embedded engineering should therefore be viewed as a selective and complementary model. It is not appropriate for every department, nor does it replace traditional hospital engineering structures or external collaborations. Nevertheless, in clinical environments where care delivery, data, technology, and research are closely intertwined, embedding engineers may help reduce the distance between biomedical innovation and everyday clinical practice. For selected departments, the future of innovation may depend not only on developing better tools, but also on placing the right expertise closer to the problems those tools are meant to solve.

## Data Availability

Not applicable.
